# Impact of Image Enhancement on the Radiomics Stability of Diffusion-Weighted MRI Images of Cervical Cancer

**DOI:** 10.7759/cureus.52132

**Published:** 2024-01-11

**Authors:** Zarina Ramli, Aishah Farizan, Nizam Tamchek, Zaharudin Haron, Muhammad Khalis Abdul Karim

**Affiliations:** 1 Department of Radiology, National Cancer Institute, Putrajaya, MYS; 2 Department of Physics, Universiti Putra Malaysia, Serdang, MYS

**Keywords:** magnetic resonance imaging (mri), diffusion-weighted imaging (dwi), clahe, standardized radiomics, cervical cancer diagnosis

## Abstract

The diffusion-weighted imaging (DWI) technique is known for its capability to differentiate the diffusion of water molecules between cancerous and non-cancerous cervix tissues, which enhances the accuracy of detection. Despite the potential of DWI-MRI, its accuracy is limited by technical factors influencing in vivo data acquisition, thus impacting the quantification of radiomics features. This study aimed to measure the radiomics stability of manual and semi-automated segmentation on contrast limited adaptive histogram equalization (CLAHE)-enhanced DWI-MRI cervical images. Eighty diffusion-weighted MRI images were obtained from patients diagnosed with cervical cancer, and an active contour model was used to analyze the data. Radiomics analysis was conducted to extract the first statistical order, shape, and textural features with intraclass correlation coefficient (ICC) measurement. The results of the CLAHE segmentation approach showed a marked improvement when compared to the manual and semi-automated segmentation methods, with an ICC value of 0.990 ± 0.005 (p<0.05), compared to 0.864 ± 0.033 (p<0.05) and 0.554 ± 0.185 (p>0.05), respectively. The CLAHE segmentation displayed a higher level of robustness than the manual groups in terms of the features present in both categories. Thus, CLAHE segmentation is owing to its potential to generate radiomics features that are more durable and consistent.

## Introduction

Cervical cancer ranks as the fourth most prevalent malignancy to affect women and the third most frequently diagnosed cancer in females worldwide [1]. At the initial stages, cervical cancer may not present any symptoms; however, as it progresses, it may manifest as bleeding after sexual intercourse, between menstrual cycles, or post-menopause, as well as an excessive amount of watery, bloody vaginal discharge [2]. Early detection of cervical cancer is associated with more favorable outcomes, with treatments such as surgery or radiotherapy proving to be effective. Unfortunately, it is estimated that the mortality rate of women aged 30 to 60 due to cervical cancer has increased significantly [3]. Low- and middle-income countries account for the majority of newly diagnosed cases and fatalities. In comparison to men, the most commonly diagnosed cancer in women is mainly comprised of two cancer sites: breast cancer (in 159 countries) and cervical cancer (in the remaining 23 out of 26 countries) [4]. The International Agency for Research on Cancer GLOBOCAN 2020 estimates that cancer incidence and mortality will rise in 2040 (28.4 million cases) [2]. This has been on the upswing due to the heightened risk factors associated with globalization and an expanding economy. It is essential for global cancer control to create a sustainable framework for the distribution of cancer prevention strategies and the delivery of cancer treatment in transitioning countries [5]. In order to effectively tackle global cancer control, a sustainable structure for the circulation of cancer prevention strategies and the provision of cancer treatment in transitioning countries needs to be established [6]. It is recommended that at least 90% of girls be vaccinated by the age of 15 in order to meet the World Health Organization's (WHO) global strategy of eliminating cervical cancer by 2030. Cervical cancer screening should begin at the age of 25 and should include primary human-papillomavirus testing every five years up until age 65 [7].

In contrast to CT and PET scans, MRI techniques enable a more comprehensive analysis of tumor biology, present greater sensitivity in soft tissue contrast, and offer a more detailed understanding of the tumor's microenvironment and microcellular activity [8]. Diffusion-weighted imaging (DWI) is a non-invasive MRI technique that allows for the observation of water molecules' movements within biological tissues [9]. Due to Brownian motion, water molecules in tissues have random motion, which can be hindered by cell components such as cell membranes and organelles. Areas of high-water diffusion appear brighter on DWI images, while areas of low-water diffusion appear darker [10]. Regions with restricted diffusion, such as those found in tumors, infections, or areas of inflammation, will appear dark on DWI images [11]. To generate DWI, a series of MR images are acquired by utilizing several diffusion-weighting gradients that are positioned in different orientations. DWI and apparent diffusion coefficient (ADC) are vital parameters that aid in measuring water molecule diffusion in tissue.

The integration of radiomics in tumor classification has been particularly evident in several studies. Two common approaches are typically used to identify the performance between observers: repeatability and reproducibility assessment [12,13]. Repeatability enables the production of consistent results by applying the same segmentation approach to the same imaging data multiple times. Meanwhile, reproducibility is used to assess the consistency of results when different segmentation techniques are employed on the same imaging data [14]. As the output varied in terms of consistency, there has been a rise in research investigating the repeatability and reproducibility of radiomics characteristics [15]. The test-retest method serves as a crucial measure of feature repeatability, derived from images of the same patient that were obtained within a relatively brief time frame [16]. The application of radiomics analysis involves the utilization of sophisticated computational techniques to transform imaging data from a specific area into a set of high-dimensional feature data [17]. This process enables the provision of valuable information regarding prognosis and the identification of potential predictive biomarkers [18,19]. However, the irregularity in both reporting and analyzing features presents a substantial hindrance to the ability to repeat and compare studies [20].

Studies have demonstrated the potential of radiomics models based on T2-weighted MRI in predicting treatment response and prognosis in various types of cancer [21,22]. These models have demonstrated high sensitivity and specificity in differentiating between different tumor types. Nevertheless, DWI-MRI also has the potential to provide valuable data for diagnosis, prognosis, and treatment response evaluation in various types of cancer [23]. The inclusion of DWI-MRI in radiomics analysis could potentially enhance the precision of predictive models due to the access it provides to additional functional and diffusion-related information [24,25]. Semi-automated segmentation techniques have been proven to be more effective than manual segmentation approaches. Furthermore, when compared to adaptive histogram equalization (AHE) and histogram equalization (HE), contrast-limited AHE (CLAHE) offers a more effective contrast enhancement option, leading to a faster volumetric measurement of the cervix segment and improved results. Thus, this study aims to assess the impact of CLAHE on DWI-MRI cervical cancer images and to compare the radiomics features in order to optimize the accuracy of clinical diagnosis.

## Materials and methods

Study design

This retrospective clinical study was approved by the Medical Research and Ethical Committee of the Ministry of Health in Malaysia on August 10, 2022, with an ethics initial approval number of NMRR ID-22-01426-OU4 (IIR). Data from all the images was acquired from the Infinitt PACS system at the Institut Kanser Negara in Putrajaya, Malaysia, within the period from January 1, 2014, to December 31, 2019. This retrospective analysis was conducted to examine the resilience of characteristics obtained from a cohort of 80 individuals diagnosed with cervical cancer. The random sampling approach was employed to obtain radiologist reports and patient demographic data, thus eliminating any biases and enabling the gathering of the most accurate data.

Scanning acquisition protocols

**Figure 1:**
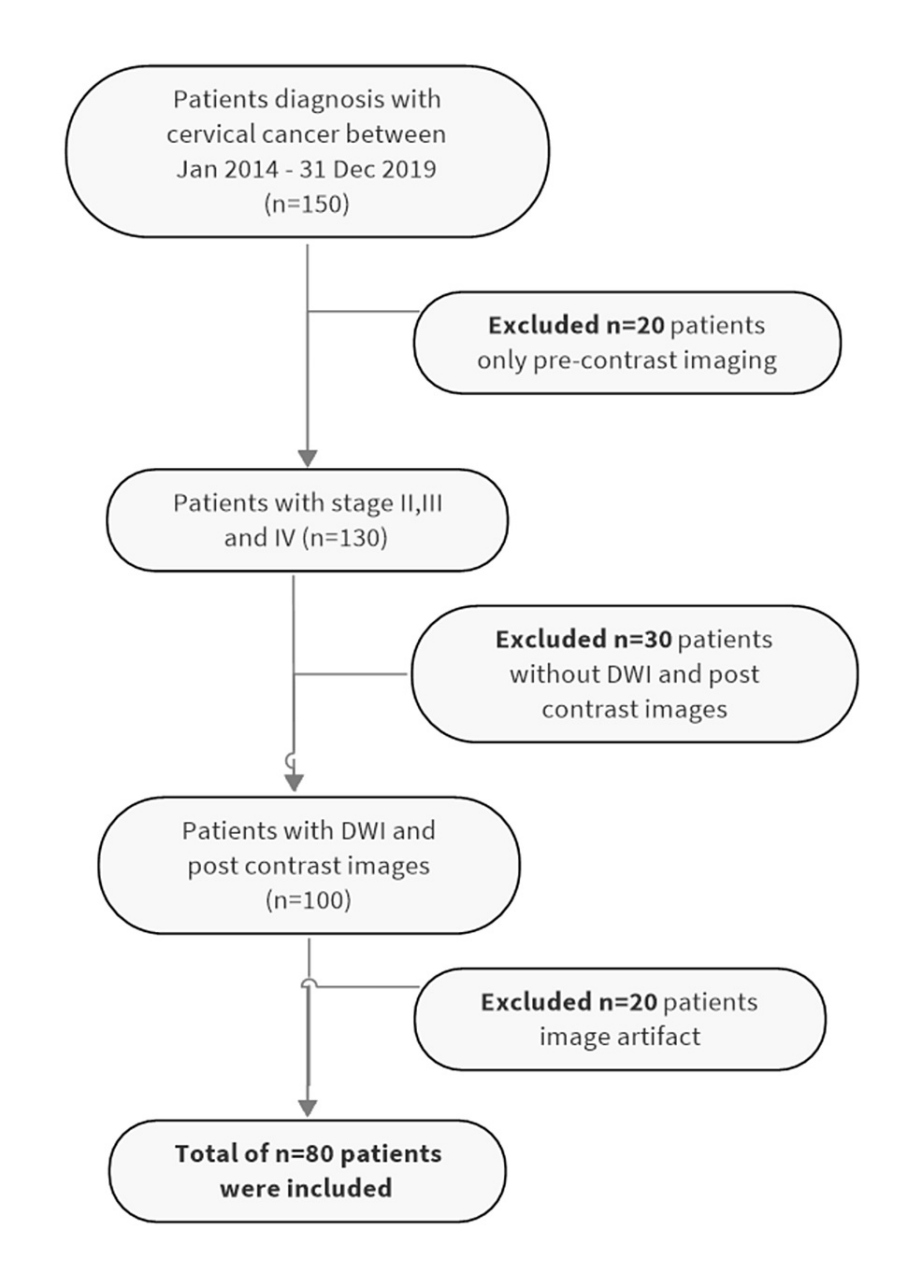
Patient selection based on inclusion and exclusion criteria DWI: diffusion-weighted imaging

Of 150 patients, only 80 patients (with a mean ± SD of age 55 ± 12.45 y/o and a weight range of 53.2 ± 5.31 kg) fulfilled the inclusion and exclusion criteria. For this study, the inclusion criteria were patients who had preoperative MRI evaluations, were diagnosed with cervical cancer stages II-IV, and had DWI pre- and post-contrast images completed. Figure 1 illustrates the selection of inclusion and exclusion criteria. As per exclusion, it comprises 20 patients exhibiting image artifacts, 30 patients who did not have both DWI and post-contrast images, and 20 patients with only pre-contrast imaging.

A comprehensive scan of the uterus and ovaries was conducted using a Siemens 3 Tesla MRI (2013, Magnetom Vario Erlangen, Germany), with the axial scan angle tailored to the specific pathology being examined. Standardized parameters were used for all sequences, including DWI sequences with a slice thickness of 3.0 mm, a base resolution of 140, a time repetition of 6300 ms, a time echo of 69.0 ms, a field of view of 220 mm, and a phase resolution of 100%. Diffusion was evaluated using pre-established b-values at B:50, B:400, and B:900, and the results were categorized and differentiated into Stage II, Stage III, and Stage IV.

Feature extraction of cervical cancer DWI-MRI

In this study, a single-blind design was employed, with the exception of the researcher, who had access to the patient report. By randomly selecting a sample of individuals with stages II-IV cervical cancer diagnoses, two senior radiologists with more than 10 years of experience in MRI scan reporting were asked to analyze the same set of images without being aware of the diagnosis. In order to reduce any potential bias in the process of image segmentation, the patient identities were deliberately withheld until the segmentation phase, at which point they were disclosed using a numerical sequence.

There have been three distinct groups established for the purpose of assessing the dependability characteristics generated through the segmentation process: based on texture, first-order statistics, and shape. The radiomics information was procured via the utilization of the active contour model (ACM) with MATLAB (version R2022b, MathWorks, USA) by two independent observers, who identified a volume of interest and performed semi-automated segmentation and CLAHE segmentation on two distinct instances.

The ACM approach is based on mathematical principles, specifically the classical snake’s energy minimization* I* of curve evolution* I* through *α, β, γ*.

$E(C)={{\int_{0}^{1}{\alpha \left| C' \right|}}^{2}}+\beta \left| C'' \right|dq-\gamma \int_{0}^{1}{\left| \nabla I(C) \right|dq}$

where C(q) of 0 to 1 is the planar curve with given matrices of pixels, *I*, parameters* α* and* β* is control for internal energy and *γ* specifically for external energy. Subsequently, the curve smoothing will be demonstrated by incorporating the gradient finite function with β = 0.

$E(C)={{\int_{0}^{1}{\left| C'(q) \right|}}^{2}}dq+\gamma \int_{0}^{1}{g{{(\left| \nabla I(C) \right|)}^{2}}dq}$

with *E_0_ ≠ 0*, the Euler-Lagrange is applied to fill C(0) = C_0_, the evolution of $\kappa$ is Euclidean curve with unit of inward, N now

$\frac{\partial C(t)}{\partial t}=g(I)\kappa -({{\nabla }_{g}}.)$

adapting C with level-set of u and, computed $\kappa$, gives

$\frac{\partial u}{\partial t}=g(I)(c+\kappa )\left| \nabla u \right|$

This iterative approach, which is often referred to as the snake's method, is used to divide images into sections. Subsequently, four independent observers were enlisted to compare it with manual segmentation.

Manual and semi-automated segmentation

**Figure 2:**
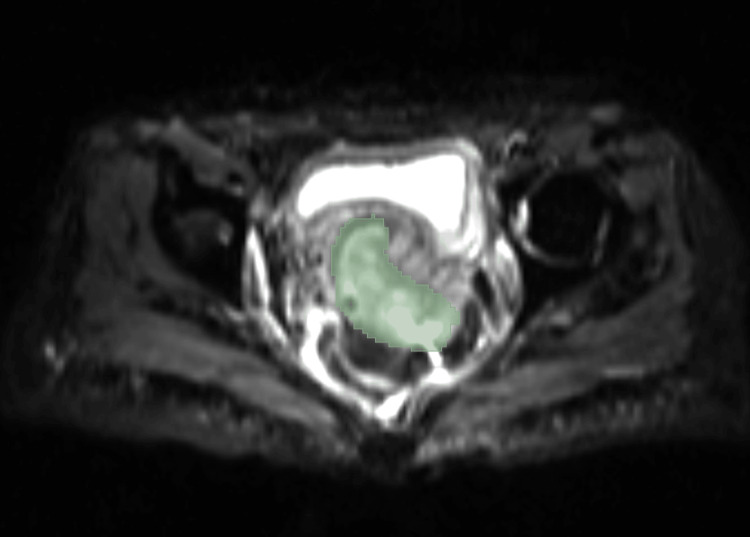
Manual segmentation on DWI-weighted MRI (axial plane stage IV) of cervical image

**Figure 3:**
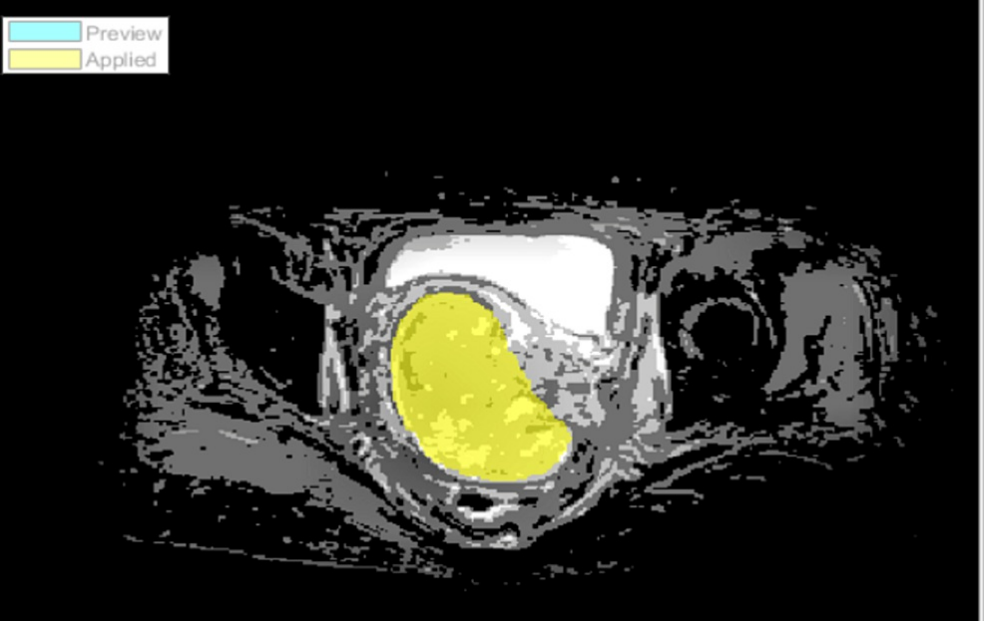
Semi-automated segmentation with CLAHE enhanced on axial plane of cervical DWI-MRI image

All segmentation processes were done by two experienced radiologists, to be used as a reference point. This image served as the basis for the manual segmentation process. Figure 2 demonstrates the manual segmentation of an axial DWI-weighted MRI cervical image. The process of semi-automatic segmentation involves the utilization of the ACM. The initial curves on an image are constructed using the active contour algorithm. Thereafter, the active contour function is employed to facilitate the growth of the curves toward the object boundaries. Figure 3 demonstrates the region of interest (ROI) of semi-automatic segmentation with CLAHE enhancement on DWI-MRI images.

CLAHE enhancement

The use of CLAHE-based semi-automated segmentation for image data enhancement requires two significant hyperparameters: clip limit (CL) and number of tiles (NT). CL is a numerical value that determines the amplification of noise, and 0.9 was chosen from the spectrum of 0 to 1 as it provided the best balance of contrast while avoiding the CL exceeding its maximum. The image data was enhanced using the CLAHE algorithm. The suspected lesion was manually marked with the region-growing algorithm, and the ROIs were divided into anterior and posterior regions and converted to binary images. To assess intra-observer reproducibility, two radiologists independently delineated the tumor area in the enhanced images twice. The iteration count for semi-automated segmentation was standardized at 100 iterations.

Statistical analysis

**Figure 4:**
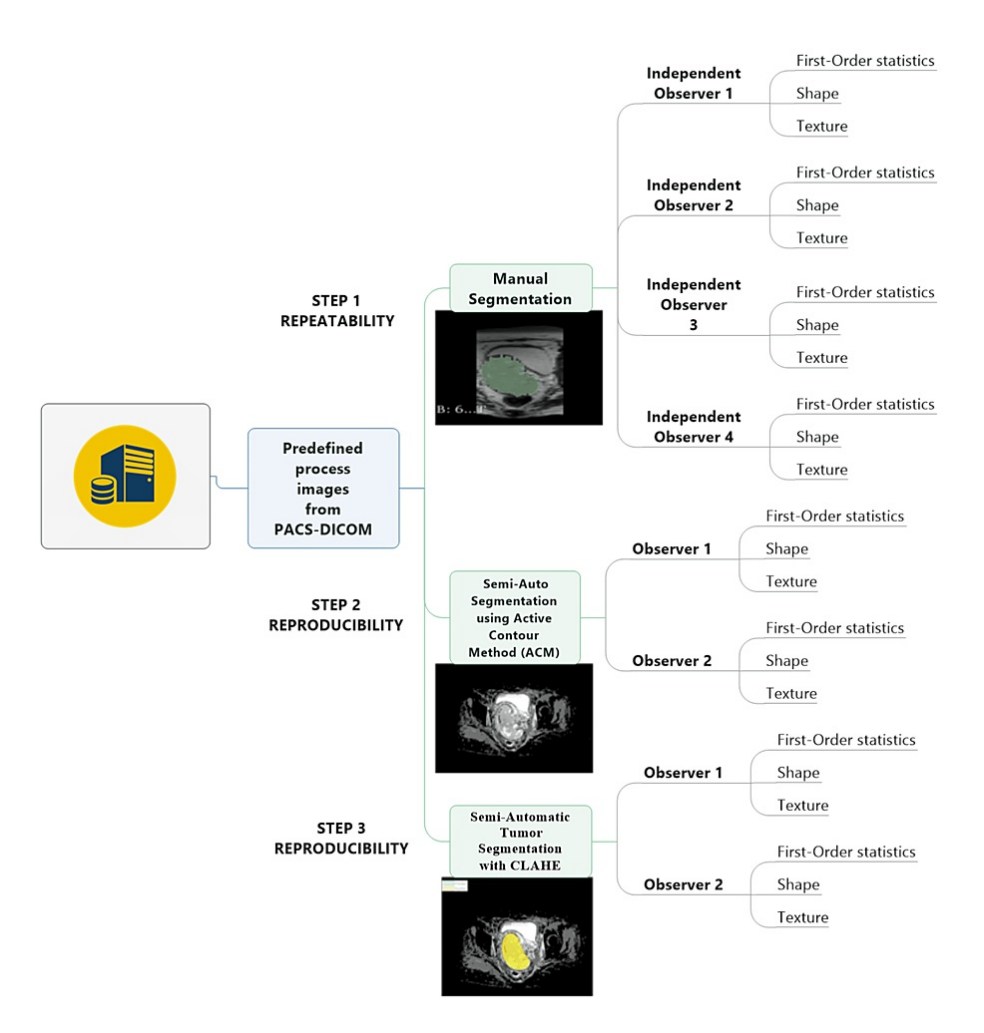
Systematic flow of manual, semi-automated, and CLAHE enhancement and analysis by the observers CLAHE: contrast limited adaptive histogram equalization

Pyradiomics was employed to eliminate any discrepancies in in-plane resolutions that could lead to confusion, thereby advancing techniques, encouraging advanced methodologies, and promoting a culture of responsibility. Table 1 presents the features that have been extracted in this study. The features were assessed using SPSS Statistics version 29.0 (IBM Corp. Released 2022. IBM SPSS Statistics for Windows, Version 29.0. Armonk, NY: IBM Corp). The research utilized three techniques, namely manual, semi-automated, and CLAHE segmentation, to extract 37 features that were subsequently categorized into three distinct groups: (1) first-order statistical features, (2) second-order statistical features utilizing gray level co-occurrence matrix (GLCM), and (3) shape features. Figure 4 illustrates the procedures involved in the manual for repeatability, semi-automated, and CLAHE segmentation for reproducibility studies.

**Table 1:** Selection of radiomics features extracted from the images GLCM: gray level co-occurrence matrix

GLCM features (GLCM_) (n=21)	First-order statistics (n=6)	Shape features (n=9)
Correlation	Entropy	Area
Contrast	Kurtosis	Major axis length
Autocorrelation	Skewness	Minor axis length
Difference variance	Energy	Perimeter
Homogeneity	Variance	Equivdiameter
Dissimilarity	Mean	Convex area
Entropy		Orientation
Energy		Solidity
Homogeneity		Eccentricity
Cluster shade		
Maximum probability		
Sum of variance		
Sum square		
Sum average		
Sum entropy		
Cluster prominence		
Information measure of correlation2		
Information measure of correlation1		
Difference entropy		
Inverse difference normalized		
Inverse difference moment normalized		

The intraclass correlation coefficient (ICC) was employed in this study to determine the correlation between two datasets. It is a numerical measure of consistency, with values ranging from 0 to 1, and is used by researchers in various fields [14,24]. The selection of a suitable ICC model is dependent on the particulars of the study, and there exist three separate models that can be opted for. This study utilized a two-way mixed effect model of ANOVA to calculate the ICC by deriving variance estimates for the repeatability and reproducibility segmentations. The ICC equation is described as follows:

$ICC(A,1)=\frac{M{{S}_{R}}-M{{S}_{E}}}{M{{S}_{R}}+(k+1)M{{S}_{g}}+{}^{k}/{}_{n}(M{{S}_{c}}-M{{S}_{E}})}$

$ICC(C,1)=\frac{M{{S}_{R}}-M{{S}_{W}}}{M{{S}_{R}}+(k-1)M{{S}_{R}}}$

where MS_C_ stands for mean square for columns, MS_R_ for mean square for rows, MS_E_ for mean square error, and MS_W_ for mean square for residual sources of variance. The letters k and n are employed to denote the number of observers and participants in the research, respectively. ANOVA was utilized to compute the ICC coefficients for the purpose of evaluating the reproducibility of intra-observer segmentation. The ICC (C,1) was applied to data collected by a single observer who segmented 80 patients at two-month intervals using two distinct segmentation methods. The Wilcoxon rank-sum test was conducted with a significance level of p<0.05 to assess the difference in reproducibility across all segmentations.

## Results

The radiomics quality score 2.0 for this study attained a score of 37.59/36 (98.6%), indicating an optimized pipeline for the ICC assessment [25]. This score provides a standardized approach for measuring consistency, reproducibility, and accuracy. The score is a testament to its quality and reliability, making radiomics features robust and ready for further analysis, thus giving accurate and meaningful insights for clinical use and research. Intra-observe analysis pertains to the measurement sets conducted by a single observer or researcher. Table 2 presents the variations in ICC when comparing manual, semi-automated segmentation, and CLAHE with regard to textural features, shape, and first-order (intensity) histogram features.

**Table 2:** Reproducibility group of features according to segmentation technique and image enhancement CLAHE: contrast limited adaptive histogram equalization, ICC: intraclass correlation coefficient

Reproducibility group	Manual	Semi-automated	CLAHE
Poor (ICC < 0.4)	38 (47.50%)	0 (0%)	0 (0%)
Fair (0.4 ≤ ICC ≤ 0.6)	26 (32.50%)	5 (6.25%)	0 (0%)
Good (0.6 ≤ ICC ≤0.75)	14 (17.50%)	20 (25.00%)	1 (1.25%)
Excellent (0.75 ≤ ICC ≤ 1)	1 (1.25%)	55 (68.75%)	79 (98.75%)

**Figure 5:**
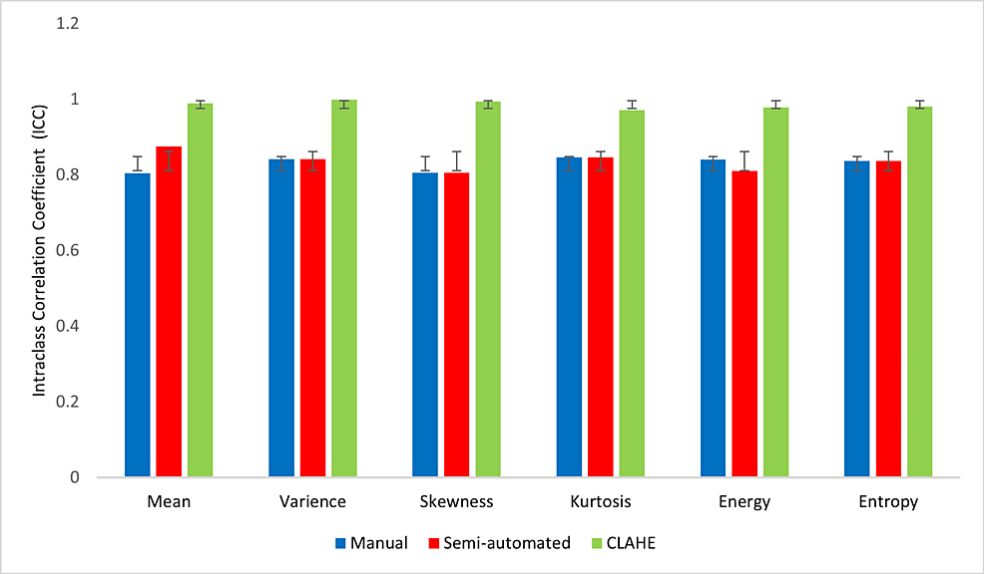
A comparison of the ICC between segmentation for first-order histogram-based features ICC: intraclass correlation coefficient, CLAHE: contrast limited adaptive histogram equalization

**Figure 6:**
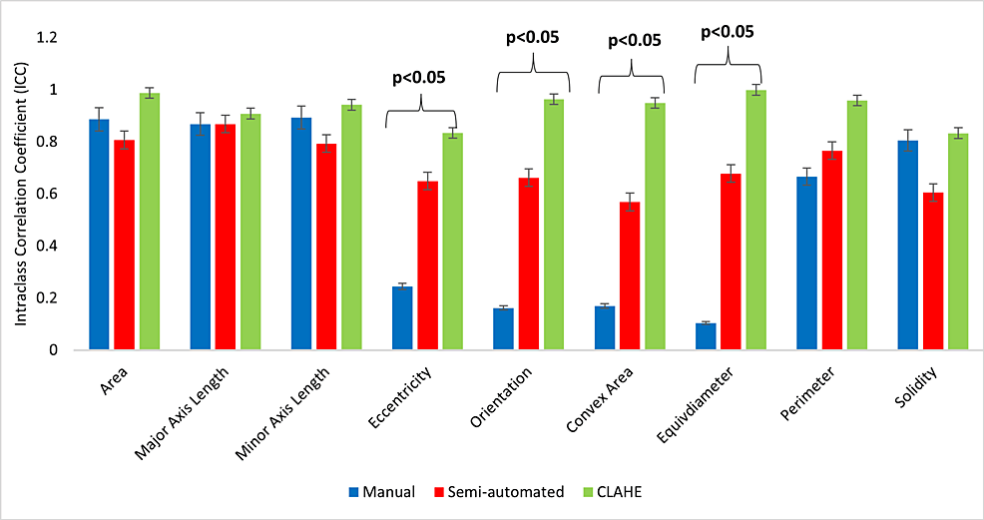
A comparison of the ICC value between segmentation for shape-based features ICC: intraclass correlation coefficient, CLAHE: contrast limited adaptive histogram equalization

**Figure 7:**
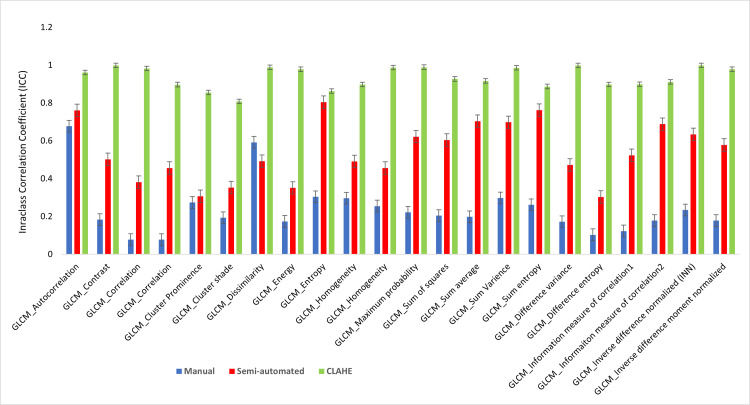
A comparison of the ICC value for the textural features ICC: intraclass correlation coefficient, CLAHE: contrast limited adaptive histogram equalization

The results indicate that the ICC for segmentation technique and image enhancement for CLAHE (ICC = 0.990 ± 0.005, p<0.05) was significantly higher than those from semi-automated segmentation (ICC = 0.864 ± 0.033, p<0.05) and manual segmentation (ICC = 0.554 ± 0.185, p>0.05). All 37 features showed excellent performance in CLAHE image data segmentation. The ICC values suggest that the image dataset with CLAHE contrast enhancement exhibited an excellent reproducibility of 98.75%, compared to 68.75% for semi-auto and 1.25% for manual. As a result of its application, CLAHE was found to improve image consistency with respect to the identified characteristics. Figures 5-7 illustrate the comparison of ICC for first-order, shape, and textural features, respectively.

Table 3 indicates that in manual segmentation for shape features (ICC= 0.554 ± 0.185) p>0.05) only four shape features demonstrated excellent reproducibility. Reproducibility for first-order (intensity) histogram features was significantly greater in images improved by CLAHE (ICC = 0.985 ± 0.01, p<0.05) and semi-automated segmentation (ICC = 0.837 ± 0.03, p<0.05). The observation results demonstrate that the application of CLAHE for image enhancement was highly consistent for all features when compared to other segmentation techniques.

**Table 3:** Variations in ICC value obtained from selected subject GLCM: gray level co-occurrence matrix, ICC: intraclass correlation coefficient, CLAHE: contrast limited adaptive histogram equalization

Features	Original	Manual	Semi-automated	CLAHE
GLCM textural features	Autocorrelation	0.677	0.761	0.961
	Contrast*	0.183	0.502	0.998
	Correlation	0.678	0.781	0.982
	Cluster prominence	0.674	0.797	0.855
	Cluster shade	0.693	0.753	0.808
	Dissimilarity	0.592	0.892	0.988
	Energy*	0.174	0.351	0.978
	Entropy	0.704	0.804	0.963
	Homogeneity	0.655	0.756	0.987
	Maximum probability	0.622	0.822	0.989
	Sum of squares*	0.204	0.604	0.927
	Sum average	0.698	0.804	0.916
	Sum variance*	0.298	0.698	0.986
	Sum entropy	0.662	0.762	0.887
	Difference variance*	0.672	0.772	0.998
	Difference entropy	0.603	0.803	0.898
	Information measure of correlation^1^	0.623	0.823	0.899
	Information measure of correlation^2^	0.678	0.888	0.911
	Inverse difference normalized	0.734	0.834	0.998
	Inverse difference moment normalized	0.878	0.978	0.979
	Mean	0.805	0.875	0.989
	Varience	0.842	0.842	0.999
First-order statistics features	Skewness	0.806	0.806	0.994
	Kurtosis	0.846	0.846	0.971
	Energy	0.841	0.811	0.979
	Entropy	0.837	0.837	0.981
	Area	0.886	0.807	0.987
	Major axis length	0.868	0.868	0.908
Shape-based features	Minor axis length	0.893	0.793	0.942
	Eccentricity*	0.244	0.649	0.834
	Orientation*	0.162	0.662	0.963
	Convex area*	0.169	0.569	0.949
	Equiv-diameter*	0.104	0.678	0.999
	Perimeter	0.666	0.766	0.959
	Solidity	0.805	0.605	0.833

**Figure 8:**
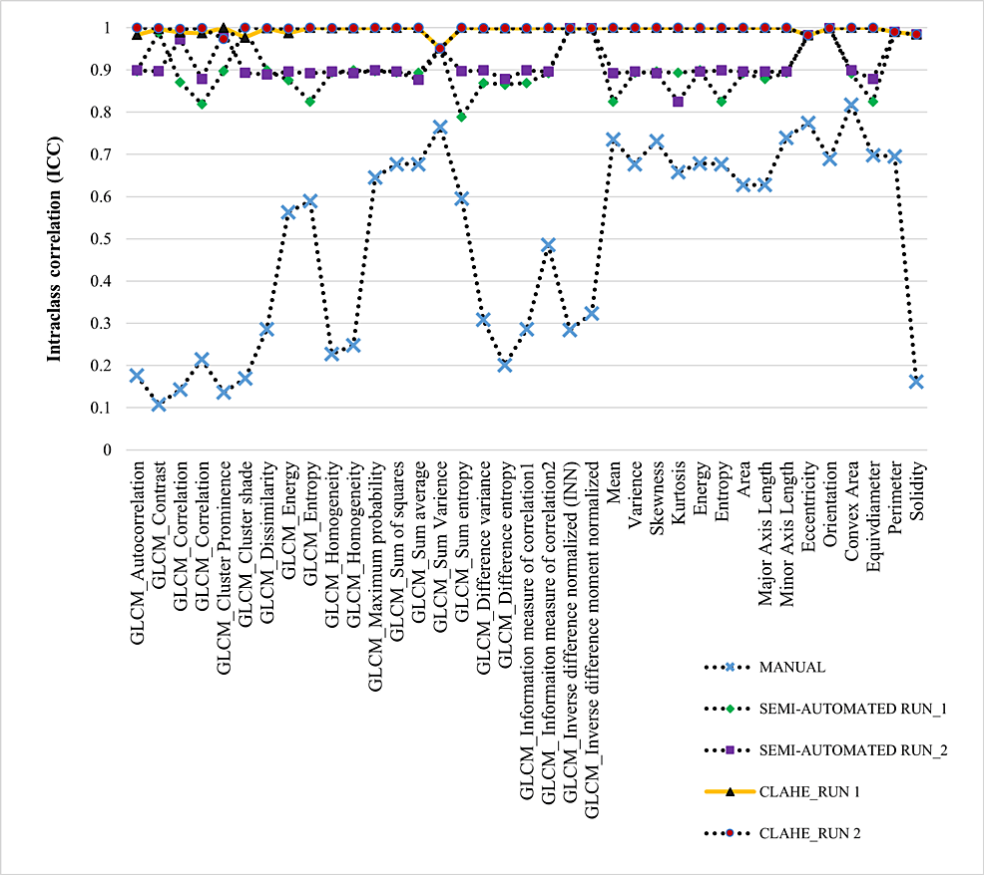
Comparison of selected ICC value among different observers GLCM: gray level co-occurrence matrix, CLAHE: contrast limited adaptive histogram equalization, ICC: intraclass correlation coefficient

The robustness of the technique was evaluated by analyzing the inter- and intra-observer ICC characteristics. Figure 8 presents the ICC value for the inter-observer segmentation group. Segmentation with CLAHE enhanced with semi-automated exhibited the highest ICC value, 0.977±0.579. This finding holds true for all 12 segmentation datasets, which are comprised of four manual sets, four semi-automated sets, and four CLAHE sets.

## Discussion

DWI-MRI plays a crucial role in radiomics for various cancer types. It quantitatively measures ADC, which reflects water diffusivity, and provides information on cell membrane integrity and tumor cellularity. Images acquired from DWI-MRI were utilized because of their ability to serve as an initial surrogate imaging biomarker for therapy responsiveness. Moreover, DWI-MRI has been identified as valuable in the evaluation of cervical cancer, providing essential information for diagnosis and treatment planning. Zhang et al. (2022) demonstrated that radiomics models with a combination of multi-parametric DWI showed high clinical value in predicting concurrent chemoradiotherapy for cervical cancer [26].

The importance of the quality of the input image cannot be emphasized enough in terms of improving the robustness of radiomics characteristics. Employing higher-resolution images can prove to be highly beneficial, as they provide a more advanced visual depiction of the segmentations, thereby resulting in enhanced outcomes [27]. In order to achieve an exact localization of the neoplasm and assess the intricate nuances of the picture, it is essential to utilize pre-processing imaging methods such as contrast enhancement. Utilization of image enhancement can lead to a substantial improvement in contrast, thereby facilitating the precise segmentation of tumors [24].

This study involved the precise semi-automated segmentation of the tumor lesion using ACM, and it was determined to have superior accuracy when compared to manual segmentation. The quantization of the tumor lesion was deemed accurate in comparison to manual segmentation based on the uniform color present within the area of interest. Radzi et al. (2021) previously showed that in order to accurately quantify the ROI, the optimal segmentation method requires images with good contrast enhancement [17,28]. In comparison to manual segmentation, it has been observed that a significant proportion of the tumor first-order features demonstrate a higher degree of reproducibility.

Upon examination of the ICC values for reproducibility between CLAHE, semi-automated segmentation, and manual segmentation, it was found that CLAHE had a higher repeatability, with a 98% rate of reproducibility. The use of semi-automated segmentation techniques with image enhancement resulted in improved reproducibility compared to manual segmentation methods. This is due to the reduced segmentation time and the use of standardized viewing settings, which aid in evaluating the intracellular changes of cervical cancer in DWI-MRI images [8,10]. The use of a standardized workstation for clinical image reporting and viewing is recommended in the research findings to ensure accurate interpretation during cervical cancer tumor segmentation. It is important to emphasize the consistent maintenance of equipment in medical settings.

Furthermore, it has been observed that the CLAHE technique can result in an amplification of the background noise present within an image. Nevertheless, it is achievable to address this issue by minimizing the extent to which the noise is amplified. By utilizing the CLAHE technique, it is possible to modify the contrast of the image to achieve a clear and enhanced appearance while avoiding any noise. One study that previously combined CLAHE and other filtration for early detection of breast cancer has proven to be effective; the noise in the background has been eliminated by the filtration, resulting in an accuracy of 97.54% [29].

The findings indicate that the application of the CLAHE technique in segmentations resulted in substantially greater ICC values compared to the semi-automatic technique and manual delineations. Although the semi-automated segmentation techniques yielded slightly lower values than the CLAHE techniques, they exhibited significantly higher levels of robustness compared to manual delineations [19,30]. The algorithm initialization enabled the semi-automated segmentation to accurately quantize the tumor region, thereby eliminating the need for observer intervention and ensuring precise segmentation. CLAHE basically enhances the HE in each region, thereby emphasizing and sharpening the elements of cervical tumors such as edges, boundaries, and contrast. The research has shown that the use of CLAHE results in increased uniformity in the extraction of radiomics features from semi-automated segmentation.

## Conclusions

This study demonstrates that the use of semi-automated CLAHE image enhancement on DWI-MRI images results in excellent reproducibility and repeatability compared to other methods. All 37 radiomics features performed exceptionally well with CLAHE, leading to improved accuracy and delineation of the subject-of-interest in DWI-MRI. When comparing the ICC values for reproducibility between CLAHE, semi-automated segmentation, and manual segmentation, it is clear that CLAHE shows superior repeatability, achieving a 98% rate of reproducibility. Therefore, it is crucial to maintain consistency in radiomics value by ensuring uniformity and optimizing the acquisition parameters of the MRI system.
